# Singular Revenge

**Published:** 1851-01

**Authors:** 


### ARTICLE X.

*Singular Revenge.*—Late one evening a person came into our office, and asked to see the Editor of the Lancet. On being introduced to our *sanctum*, he placed a bundle upon the table, from which he proceeded to extract a vesy fair and symmetrical lower extremity, which might have matched

"Atlanta's better part."

ond which had evidently belonged to a womrn. "There," said he, "is there anything the matter of that leg? Did you ever see a handsomer? What ought the man to be done with who cut it off?" On having the meaning of these interrogatories put before us, we found that it was the leg of the *wife* of our evening visitor. He had been accustomed to admire the lady's leg and foot, of the perfection of which, she was, it appeared, fully conscious. A few days before, he had excited her anger, and they had quarrelled violently, upon which she left the house, declaring she would be revenged on him, and that he should never see the objects of his admiration again. The next thing he heard of her was, that she was a patient in **** Hospital, and had had her leg amputated. She had declared to the surgeons that she suffered intolerable pain in the knee, and had begged to have the limb removed, a petition the surgeons complied with, and thus became the instrument of her absurd and self-torturing revenge upon her husband!—*London Lancet.*

